# The relationship between use of complementary and alternative medicine and health literacy in chronically ill outpatient cases: a cross-sectional study in southeastern Iran

**DOI:** 10.3389/fpubh.2023.988388

**Published:** 2023-04-27

**Authors:** Mahlagha Dehghan, Mahla Mohebi Rad, Leyla Ahmadi Lari, Behnam Ghorbani-nejad, Milad Mohebi-Rad

**Affiliations:** ^1^Department of Critical Care Nursing, Razi Faculty of Nursing and Midwifery, Kerman University of Medical Sciences, Kerman, Iran; ^2^Student Research Committee, School of Nursing and Midwifery, Kerman University of Medical Sciences, Kerman, Iran; ^3^Department of Anesthesiology, School of Nursing, Larestan University of Medical Sciences, Larestan, Iran; ^4^Department of Toxicology and Pharmacology, Faculty of Pharmacy, Kerman University of Medical Sciences, Kerman, Iran; ^5^School of Medicine, Rafsanjan University of Medical Sciences, Rafsanjan, Iran

**Keywords:** complementary medicine, health literacy, chronic outpatients, alternative medicine, integrative medicine

## Abstract

**Background:**

Chronically ill outpatient cases use a variety of complementary and alternative medicines due to their diseases and therapeutic complications. Chronic condition, quality of life, and health literacy all affect the use of complementary medicine among chronically ill outpatient cases. Health literacy helps patients make fully informed decisions about the use of complementary and alternative medicine. This study aimed to investigate the relationship between complementary and alternative medicine and health literacy in chronically ill outpatient cases.

**Method:**

This cross-sectional analytical-descriptive study was conducted on 400 chronically ill outpatient cases referred to medical centers affiliated to Kerman University of Medical Sciences. Convenience sampling was used. Research tools included the complementary and alternative medicine questionnaire and the health literacy questionnaire. SPSS25 was used to analyze data.

**Results:**

The mean use of complementary and alternative medicine in a recent year was 16.75 ± 7.89, which was lower than the mid-point of the questionnaire (84). Prayer, medicinal plants, vitamin supplements, music therapy, and art therapy were the mostly used complementary and alternative medicine methods. The most common reasons for using complementary medicine were to reduce physical complications and improve anxiety and stress. The mean satisfaction with the use of complementary and alternative medicine was 34.96 ± 6.69. The mean health literacy score was 67.13 ± 19.90. Decision-making and the use of health information had the highest mean score among the dimensions of health literacy, whereas reading skills received the lowest mean score. We found a significant and direct relationship between the use of complementary and alternative medicine, health literacy, and all its dimensions.

**Conclusion:**

The study results showed that health literacy predicted the use of complementary and alternative medicine. Health education and promotion programs may be useful for improvement of health literacy in the community.

## Introduction

The 21st century witnesses an increasing rate of chronic diseases in healthcare systems around the world (1). The term “chronic diseases” refers to conditions that cause variation or damage in structure or function of any part of the body. They last for a long time and may get worse over time (2). Chronic diseases are currently the cause of 40% of all deaths in the world and account for 47% of the global burden of diseases (3). According to Barbosa et al. (4), chronic diseases in the USA account for about 80% of all deaths (4). According to the report of the World Health Organization, chronic diseases are the leading cause of death in Iran (70%) (5). To control chronic diseases and prevent their complications, it is necessary to follow a healthy lifestyle, change high-risk behaviors such as overeating, smoking, immobility, adhere to mental health principles, and avoid stressful situations (6). Despite the progress in modern medicine, a strong focus has recently been on the use of complementary and non-pharmacological treatments in the healthcare system (7). Today, complementary and alternative medicine is a common method to control symptoms and strengthen the condition of chronically ill patients (8). According to the World Health Organization (WHO), complementary and alternative medicine is knowledge, skills, and practices based on theories, beliefs and experiences indigenous in different cultures used to maintain health, prevent, improve or treat physical and mental problems (9). Complementary medicine is divided into biological treatment methods, including herbal medicines, vitamins and food supplements and non-biological methods, including acupuncture, hydrotherapy, massage and music therapy (10). Based on the World Health Report (2017), complementary and alternative medicine is related to lifestyle and meets health needs due to its potential application in the prevention and management of chronic diseases (11). Ince et al. (12) in Turkey reported that 27% of the chronically ill patients used herbal remedies (6.63%) and wet cupping (4.25%). Level of education had a significant correlation with herbal remedies and patients used different types of complementary medicine as their disease progressed (12).

Today, the use of CAM has increased among Iranian patients to reduce complications (13). Traditional Iranian medicine is a type of CAMs based on four temperaments. Temperament is an essential element of traditional Iranian medicine that uses all the elements in nature, including herbal, mineral and animal materials (14). According to a study, more than half of the Iranians use at least one type of complementary and traditional medicine (13).

Chronic condition, socio-economic status, education, income, race, health-related behaviors, quality of life, and health literacy of these patients affect their use of complementary medicine (15). Clients may not assess various treatment techniques if they lack health literacy, which is the best predictor of health status. The use of complementary and alternative medicine is increasing, and health literacy helps make a fully informed decision about the use of complementary medicine (16, 17). It is necessary to understand the nature and impact of health literacy on promoting health and preventing and treating chronic diseases (18). Chronically ill patients need health literacy to manage their disease symptoms and problems and improve their quality of life; health literacy is known as an important indicator of healthcare outcomes (19). Non-adherence to treatment and limited health literacy can reduce the perception of treatment methods, increase medication errors and the mortality rate (20). Health literacy refers to the cognitive and social skills that determine motivation and ability of individuals to gain access to health information (21). Jayasinghe et al. (22) in Australia showed that patients with limited health literacy had insufficient physical activity, were overweight, had poor physical and mental health, and had history of smoking (22). Brooks et al. (23) in the UK found that one out of every three older people had insufficient health literacy, meaning that they had difficulty reading and understanding basic medical information such as the medication dosage (23).

Improved health literacy is associated with better health outcomes, including changes in chronic disease risk (24). Charoencheewakul et al. (25) in Thailand studied health literacy and the use of complementary and alternative medicine among patients with type 2 diabetes and showed that 30.89% of them used complementary medicine. Patients with enough health literacy in the use of complementary and alternative medicine had 2.64 times higher odds of complementary and alternative medicine. Health literacy, economic status, and diabetes control had a great impact on the use of complementary and alternative medicine (25). Smith et al. (17) in Australia showed that 75% of the older people used complementary medicine. The most common source of information on the use of complementary medicine was general practitioners, and the use of complementary medicine had no significant relationship with the health literacy of people (17). Sharoni et al. (26) studied the use of complementary and alternative medicine and health literacy among patients and showed that 35.6% of them used complementary medicine and 27% of them were satisfied with the use of complementary medicine. Patients had a high literacy level in the use of complementary medicine, and a positive relationship was available between health literacy and age. They also showed that health literacy of women was significantly higher than that of men and older women had better health literacy in the use of complementary medicine (26).

A review of the literature shows that healthcare workers are increasingly studying about the CAM as one of the treatment methods (27, 28). It is very important to evaluate factors affecting health literacy in chronically ill outpatient cases. Some studies reported a significant relationship between the use of complementary medicine and health literacy (15, 25, 29), but other studies suggested no significant relationship between these two variables (17, 30), and we found few studies on the use of complementary medicine and health literacy; therefore, this study aimed to examine the relationship between the use of complementary medicine and health literacy in chronically ill outpatient cases in Kerman, Iran in 2021.

## Materials and methods

### Study type and setting

This cross-sectional descriptive-correlational study was conducted on chronically ill outpatient cases (cardiovascular, diabetes, hypertension, cancer, thyroid and etc.) referred to Besat Clinic 1, Besat Clinic 2, Javad Al-Aemeh Clinic, as well as Shafa, Bahonar, Afzalipur hospitals in Kerman from July to mid-September 2021. Clinics are the main referrals for chronically ill outpatient cases.

### Study sampling and sample size

The target population of this study included all outpatient cases with chronic diseases. The sample size in the present study was obtained using the following sample size formula (power = 80%, *Z* = 1.96, *d *= 0.05, *p*&*q* = 0.5).

$n=\frac{Z^{2}pq}{d^{2}}$ = $\frac{{\left(1.96\right)}^{2}0.5\times 0.5}{{\left(0.05\right)}^{2}}=384.16.$

The inclusion criteria were individuals aged 18–65 years old with good mental health, speech, vision and hearing, who had been diagnosed with chronic disease for at least 1 year (31). The exclusion criterion was incomplete questionnaires. Convenience sampling method was used. Considering the dropout rate, 450 eligible participants completed the health literacy and CAM questionnaires, but 40 questionnaires were removed from the study due to data missing. In addition, 10 participants declined to participate in the study. Therefore, the effective response rate was 88.88%.

### Study tools

This study used three questionnaires of the demographic and background information, the complementary and alternative medicine, and the health literacy.

The demographic and background information questionnaire included gender, age, marital status, place of residence, education level, employment status, insurance, and income. The clinical information of the patients also included body mass index, type of chronic disease, and duration of the disease.

### Complementary and alternative medicine questionnaire

This is a researcher-conducted questionnaire with 24 questions about the use of various types of complementary medicine (herbs, dry cupping, wet cupping, massage, diet, acupuncture, acupressure, hydrotherapy, aromatherapy, vitamin supplements, reflexology, touch therapy, homeopathy, energy therapy, and leech therapy, various methods of relaxation such as yoga and meditation, prayer, hypnosis, psychotherapy, group therapy, art therapy, music therapy, mindfulness). The questionnaire was scored on an 8-point Likert scale ranging from never to every day. In addition, the reasons for adopting complementary medicine were measured using three options: symptom reduction, anxiety reduction, and others. The minimum score was zero, while the maximum was 168, with a higher score indicating higher use of complementary and alternative medicine. In addition, 10 items were used in this questionnaire to measure the level of satisfaction with complementary medicine on a five-point Likert scale (completely satisfied = 4, satisfied = 3, no idea = 2, dissatisfied = 1, completely dissatisfied = 0), with the highest score being 40 and the lowest score being zero.

The new and related scientific books, articles, similar researches and websites were used to determine the content of the questionnaire (32). Then, the content validity was used to determine the questionnaire’s validity. Ten faculty members reviewed the questionnaire and confirmed the content validity index of 0.98. In addition, the test–retest method was used to determine the reliability. The questionnaire was given to 50 individuals who met the inclusion criteria and their scores were calculated. The same individuals recompleted the questionnaire after 2 weeks. The intra-class correlation coefficient (ICC) was 0.71.

### Health literacy questionnaire

The questionnaire used in this research was the revised and final version of the health literacy questionnaire with two parts: the first is information about the subjects and the second is the main items, including reading skills (4 items), access (6 items), comprehension (7 items), assessment (4 items), decision-making and use of health information (12 items). The raw score of each subscale was obtained from the sum of the scores. Then, the following formula was used to convert this score to a range of 0 to 100.

((Minimum raw score – obtained score)/(maximum score - minimum score)) × 100.

To calculate the total score, the scores of the subscales (ranging from zero to 100) were added up and divided by the number of subscales (5). Finally, the health literacy score was divided into insufficient (0–59), borderline (60–74), and sufficient (75–100) levels based on the cutoff points of 59–74. Ali Montazeri et al. (33) in Iran evaluated the questionnaire’s validity and reliability and determined the questionnaire’s validity using content validity and confirmed the reliability using Cronbach’s alpha of 0.72–0.89 (33).

### Data collection

We started sampling after the approval of the project and its code of ethics, and presentation of the cover letters to the clinics and hospitals. The researcher tried not to disrupt the treatment course of patients and visited them when they were in a good mental and psychological condition, and had enough time to answer the questions. The data collection tool was questionnaires. The researcher interviewed with the participants to complete the questionnaires.

### Ethical considerations

After obtaining the code of ethics (IR.KMU.REC.1400.120) from the Ethics Committee of Kerman University of Medical Sciences, the researcher referred to the research setting and obtained the necessary permission to conduct the study. First, the research process, its objectives, and the confidentiality of the information were explained to the research units and their written informed consent was obtained.

### Data analysis

SPSS-25 was used to analyze data. Frequency, percentage, mean and standard deviation were used to describe the study variables. Pearson correlation coefficient was used to determine the relationship between the use of complementary and alternative medicine, health literacy and its dimensions. In order to investigate the relationship between the use of complementary and alternative medicine and health literacy based on background variables, independent *t-*test and ANOVA were used; otherwise, Mann–Whitney and Kruskal-Wallis tests were used. Stepwise regression was used to determine the predictors of the use of complementary and alternative medicine. A significance level of <0.05 was considered.

## Results

Four hundred patients completed the questionnaires. The mean age of the samples was 53.33 ± 10.67 (minimum = 19 and maximum = 65). Most of the samples were female, married, unemployed/housewives, and lived in Kerman. They had lower secondary education and suffered from diabetes (Table 1).

**Table 1:** Participants’ characteristics and their relations with the use of complementary and alternative medicine and health literacy.

Variable	*N* (%)	Use of CAM		Statistical test (*p*-value)	Health literacy		Statistical test (*p*-value)
		Mean	SD		Mean	SD	
Age (yr.)							
19–30	17 (4.3)	16.41	10.60	H = 5.03 (0.28)	74.93	13.44	H = 29.16 (< 0.001)
31–40	42 (10.5)	19.10	9.74		78.98	15.55	
41–50	70 (17.5)	15.57	7.65		69.40	20.16	
51–60	156 (39.0)	17.15	7.50		65.09	20.0	
>60	115 (28.7)	16.11	7.24		63.04	19.62	
Gender							
Female	300 (75.0)	16.98	8.07	t = 1.04 (0.30)	65.41	20.48	Z = −2.93 (0.003)
Male	100 (25.0)	16.04	7.31		72.29	17.11	
Marital status							
Single	22 (5.5)	15.45	9.37	*F* = 0.48 (0.62)	80.34	10.08	H = 24.43 (< 0.001)
Married	330 (82.5)	16.73	7.90		67.81	19.67	
Divorced/widow	48 (12.0)	17.44	7.14		56.41	20.13	
Place of residence							
Kerman city	255 (63.7)	17.65	7.86	*F* = 5.82 (0.003)	69.48	18.17	H = 7.34 (0.02)
Kerman province	127 (31.8)	15.52	7.58		62.86	21.57	
Other provinces	18 (4.5)	12.61	8.36		64.05	26.12	
Level of education							
< Diploma	238 (59.5)	15.51	7.31	*F* = 7.46 (0.001)	57.94	18.91	H = 147.31 (< 0.001)
Diploma	122 (30.5)	18.64	8.17		79.46	11.97	
Academic	40 (10.0)	18.32	9.01		84.25	11.87	
Job							
Unemployed	280 (70.0)	16.24	8.08	*F* = 2.07 (0.13)	63.04	20.15	H = 43.60 (< 0.001)
Employed	53 (13.2)	18.34	8.36		77.02	16.15	
Retired	67 (16.8)	17.61	6.38		76.42	15.31	
Insurance							
Yes	370 (92.5)	17.12	7.74	t = 3.35 (0.001)	68.0	19.44	t = 3.10 (0.002)
No	30 (7.5)	12.17	8.41		56.42	22.58	
Monthly income (million Toman)							
Nothing	53 (13.3)	13.94	7.43	*F* = 3.15 (0.008)	56.56	21.42	H = 55.02 (< 0.001)
<2	84 (21.0)	15.62	7.22		59.08	20.23	
2–3	31 (7.8)	15.48	7.22		65.87	20.31	
3–4	59 (14.8)	17.42	7.32		69.37	19.39	
4–5	45 (11.3)	17.69	9.81		67.29	18.82	
>5	128 (32.0)	18.31	7.82		76.01	14.99	
Body mass index							
<18.5	20 (5.0)	15.30	8.47	*F* = 1.36 (0.25)	68.20	20.54	*F* = 0.26 (0.90)
18.5–24.9	128 (32.0)	17.81	8.55		68.46	20.65	
25–29.9	168 (42.0)	16.69	7.70		66.22	20.33	
30–34.9	67 (16.8)	15.91	6.92		66.45	17.64	
≥35	17 (4.2)	14.29	6.96		67.53	19.07	
Type of chronic disease							
Cardiovascular	50 (12.5)	16.24	8.58	*F* = 0.81 (0.54)	66.79	22.50	H = 9.65 (0.09)
Diabetes	185 (46.3)	17.11	6.68		67.38	18.61	
Hypertension	80 (20.0)	16.81	7.04		61.87	22.0	
Cancer	30 (7.5)	14.60	6.62		73.13	18.44	
Thyroid	21 (5.2)	15.62	10.21		71.64	21.62	
Others	34 (8.5)	17.97	9.28		70.60	15.42	
Duration of disease (yr.)							
1–5	153 (38.3)	15.98	7.86	*F* = 1.72 (0.15)	67.15	20.58	*F* = 3.52 (0.008)
5–10	124 (31.0)	17.89	8.64		70.29	17.73	
10–15	57 (14.2)	16.02	7.14		63.36	20.86	
15–20	40 (10.0)	15.85	6.69		59.07	19.31	
>20	26 (6.5)	18.81	7.64		72.61	20.53	

CAM, Complementary and Alternative Medicine; SD, Standard deviation; H, Kruskal–Wallis H; F, Analysis of variance; *t*, independent *t* test; Z = Mann–Whitney U.

**Figure 1:**
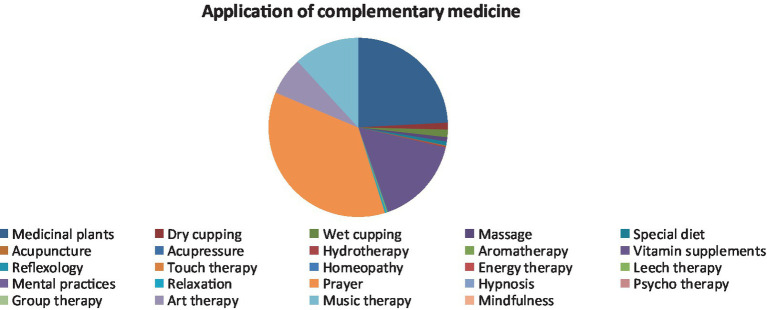
Use of complementary and alternative medicine.

Prayer, herbal remedies, vitamin supplements, music therapy, and art therapy were the most commonly used types of complementary and alternative medicine. Nobody used acupuncture, acupressure, hydrotherapy, aromatherapy, reflexology, touch therapy, homeopathy, energy therapy, relaxation, leech therapy, hypnosis, psychotherapy, group therapy, mindfulness, and mental practices (Figure 1). The most common reason for using prayer, music therapy, and art therapy was to reduce stress and anxiety. The most common reasons for using herbal remedies were to reduce complications and physical symptoms, stress, and anxiety, and the most common reason for using vitamin supplements was to reduce complications and physical symptoms (Table 2). The mean satisfaction with the use of complementary and alternative medicine was 34.96 ± 6.69 (minimum = 2 and maximum = 40), which was higher than the mid-point of the questionnaire (20).

**Table 2:** Discription of the reasons for use of different types of complementary and alternative medicine.

Type of CAM	Mean	SD	Reason for use (n/%)		
			Reducing physical symptoms	Alleviating stress and anxiety	Both
Medicinal plants	4.05	3.02	34 (8.5)	56 (14.0)	171 (42.8)
Dry cupping	0.21	0.53	56 (14.0)	1 (0.3)	–
Wet cupping	0.23	0.53	66 (16.5)	1 (0.3)	–
Massage	0.13	0.60	25 (6.3)	–	2 (0.5)
Special diet	0.12	0.76	8 (2.0)	3 (0.8)	2 (0.5)
Acupuncture	0.02	0.16	5 (1.3)	–	–
Acupressure	0.008	0.11	2 (0.5)	–	–
Hydrotherapy	0.04	0.34	7 (1.8)	–	–
Aromatherapy	0.005	0.10	–	1 (0.3)	–
Vitamin supplements	2.68	3.08	216 (54.0)	1 (0.3)	1 (0.3)
Reflexology	0.02	0.35	2 (0.5)	–	–
Touch therapy	0.0	0.0	–	–	–
Homeopathy	0.0	0.0	–	–	–
Energy therapy	0.0	0.0	–	–	–
Leech therapy	0.03	0.20	8 (2.0)	–	–
Mental practices	0.005	0.07	–	2 (0.5)	–
Relaxation	0.05	0.48	1 (0.3)	5 (1.3)	–
Prayer	6.06	2.29	2 (0.5)	359 (89.8)	1 (0.3)
Hypnosis	0.008	0.15	–	–	–
Psycho therapy	0.02	0.19	1 (0.3)	2 (0.5)	–
Group therapy	0.008	0.09	1 (0.3)	2 (0.5)	–
Art therapy	1.13	2.38	1 (0.3)	104 (26.0)	–
Music therapy	1.98	2.87	–	202 (50.5)	–
Mindfulness	0.005	0.10	–	–	–

CAM, Complementary and Alternative Medicine, SD, Standard deviation.

The mean score of using different types of complementary and alternative medicine in the recent year was 16.75 ± 7.89, which was lower than the mid-point of the questionnaire (84). The mean score of health literacy was 67.13 ± 19.90, which was higher than the mid-point of the questionnaire (34). Among different dimensions of the health literacy, decision-making and use of health information received the highest mean score, while reading skills received the lowest mean score (Table 3).

**Table 3:** Discription of use of complementary and alternative medicine and health literacy and their relations.

Varible	Minmum	Maximum	Mean	SD	Use of CAM	
					*r*	*p*-value
Use of CAM	0	45	16.75	7.89	–	–
Health literacy	9.58	99.17	67.13	19.90	0.37	< 0.001
Reading	0	100	53.47	32.54	0.28	< 0.001
Access	0	100	73.33	19.19	0.32	< 0.001
Comprehension	0	100	70.37	29.22	0.33	< 0.001
Evaluation	0	100	58.84	26.60	0.32	< 0.001
Decision-making	14.58	100	79.65	15.54	0.22	< 0.001

CAM, Complementary and Alternative Medicine; SD, Standard deviation; r, Pearson correlation.

We found a significant relationship between place of residence, education level, insurance, income, and the use of complementary and alternative medicine. People living in Kerman used more types of complementary and alternative medicine than people living in other provinces (*p* = 0.003). In addition, individuals with a diploma or higher education used complementary and alternative medicine more than individuals who had lower secondary education (*p* = 0.001). Insured individuals used complementary and alternative medicine more than uninsured ones (p = 0.001). Furthermore, individuals with higher incomes used complementary and alternative medicine more (*p* = 0.008; Table 1). Except for the type of chronic disease and body mass index, other background variables were significantly related to health literacy (Table 1).

In order to determine the predictors of the use of complementary and alternative medicine, we imported all significant variables in the bivariate analysis into the stepwise regression model. Complementary and alternative medicine was a dependent variable, while health literacy, place of residence, level of education, insurance, and income were independent variables. The results showed that only health literacy and insurance predicted the use of complementary and alternative medicine (Table 4).

**Table 4:** Multiple regression analysis summary for the use of complementary and alternative medicine.

Variable	Unstandardized coefficients		Standardaized coefficients	*T*	*p*-value	95% Confidence for B	
	*B*	Standard error	Beta			Lower bound	Upper bound
Constant	4.22	1.69		2.49	0.01	0.89	7.55
Health literacy	0.14	0.02	0.36	7.59	< 0.001	0.10	0.18
Insurance	3.32	1.4	0.11	2.37	0.02	0.57	6.07

Adjusted *R*^2^ = 0.146, *F* = 35.20, *p* < 0.001.

## Discussion

The present study aimed to examine the relationship between the use of complementary medicine and health literacy in Iranian chronically ill outpatient cases. The results indicated the low mean use of complementary and alternative medicine in the last year. Ince et al. (12) supported our results and showed that only a small percentage of chronically ill patients used herbal remedies (12). Rafi et al. (35) reported that Bangladeshi patients with diabetes used complementary medicine less than those in other Asian countries (35). Chukasemrat et al. (36) in Thailand found that only a quarter of female cancer patients used herbal remedies (36) due to uncertainty about the effectiveness and lack of access to complementary medicine, different socio-cultural orientations, beliefs and attitudes of patients and healthcare systems, and access to modern medicine (35). Hasan et al (37) disagreed with us and showed a high percentage of complementary medicine use in chronically ill outpatient cases participating in this study (37) because they were interested in trying new alternative treatments regardless of conventional treatment and family history of CAM use. In addition, about 78% of them were satisfied with the use of CAM.

The results also showed that prayer, herbal remedies, vitamin supplements, music therapy, and art therapy were the most commonly used types of complementary and alternative medicine. Prayer gave patients a deep peace of mind and caused them to adjust to the disease process and course of treatment so that they could continue their treatment. As Iran is a religious country and the dominant religion in Iran is Islam, most people pray three times a day according to their religious belief. Therefore, the use of prayer by the participants in this study was completely predictable. Ibrahim et al. (38) studied patients with hypertension in Iraq and found that most of them used herbal remedies, diets, vitamins and nutritional supplements (38). Radwan et al. (39) in the UAE showed that patients with type 2 diabetes used local foods, herbal remedies, spiritual therapy, and vitamin supplements (39). Rafi et al. (35) demonstrated that the most commonly used complementary medicine was herbal remedies (35). The different use of complementary and alternative medicine in different studies can be attributed to different treatments in different geographical areas (40), as well as different religious and cultural beliefs (41). One of the reasons people use herbal remedies more than others methods was that they were widely available. Herbal medicine companies make these products more available to people, which are easy to use. In addition, the general policy of Iran encourages people to use indigenous herbal treatments for economic reasons and reduced dependency (42).

According to the results, the most common reasons for using complementary medicine were to reduce physical complications and improve anxiety and stress. Chronically ill patients used complementary or alternative medicine to meet their needs due to the difficulty of their treatment course. Shahjalal et al. (43) in Bangladesh indicated that chronically ill patients used complementary medicine because it was easy to use and cost effective, prevented adverse side effects, and managed chronic diseases (43). Babayiğit et al. (44) in Turkey believed that chronically ill patients used complementary medicine due to its faster recovery and fewer complications than modern medicine (44). Johny et al. (45) in Malaysia believed that chronically ill patients used complementary medicine to prevent the complications of their disease. They used diet and naturopathy to strengthen and maintain their health status (45). However, Jameson et al. (46) found that Indian patients referred to a rheumatology clinic did not use yoga and acupuncture (46) because they were unaware of the complementary medicine methods and had painful acupuncture.

According to the study results, the majority of individuals were completely satisfied with the use of different kinds of complementary medicine because they had no side effects and were safer than modern medicine. Some studies in this field confirmed the results of the present study. Kaur et al. (47) in Malaysia, Bhalerao et al. (48) in India, and Erku et al. (34) in Ethiopia showed that 40–91.8% of the patients with epilepsy, rheumatoid arthritis, AIDS, diabetes, and hypertension were satisfied with the positive effect of complementary medicine on their health (34, 47, 48). Fewer side effects, better relationship between doctor and patient, longer duration of care, and variety of methods were among the reasons for patients’ satisfaction with complementary medicine (49).

The study results showed that the total mean score of health literacy and its dimensions in chronically ill outpatient cases was borderline. This result was consistent with the results of Naimi et al. in Iran and Tung et al. in Taiwan (50, 51). Jandorf et al. (52) in Denmark showed limited health literacy in most of the chronic patients (52). These results can be due to the cultural difference, the level of education of the research samples (at least high school degree) and sample size. Chronically ill patients are at risk of inadequate care and poor outcomes such as lack of awareness of the disease, poor care, increased number of admissions, and mortality. Therefore, patients need adequate health literacy to use healthcare systems and make good decisions about their health (51).

Regarding health literacy, decision-making and use of health information received the highest mean score, which was consistent with the research on patients with hypertension conducted by Mohammadpour et al. (53). One study also showed that the ability to make decisions, understand and communicate with information received the highest score (54). Decision-making requires skills such as self-awareness. Interpersonal relationship, critical thinking, and creative problem solving are influenced by many factors, including personality, environmental factors, information, importance and transparency of the issue, reward and punishment, correct perception of the situation, values and beliefs. Therefore, decision-making is influenced by both internal and external factors.

The reading skill dimension received the lowest mean score, which was in line with the research on cancer patients conducted by Bolourchifard et al. (55). Studies showed that the reading dimension of health literacy was sufficient in high resource countries but insufficient in low resource countries (56). One of the main obstacles to health literacy is the difficulty in reading materials and communicating verbally with healthcare providers. Reading requires a lot of attention and concentration because some people, even with academic education, are not able to read orders and prescriptions due to their illegibility (55). Healthcare providers should give patients easy-to-understand educational materials and all the information they need, meaning that patients with improved reading skills may utilize CAM for health promotion or health education purposes.

The study results showed a significant and direct relationship between the use of different kinds of complementary and alternative medicine, health literacy, and all its dimensions, meaning that health literacy predicted the use of complementary and alternative medicine. Charoencheewakul et al. study (25) among Thai patients with diabetes, Barnes et al. systematic review (29) on pregnant and lactating women, Dişsiz et al. study (57) on Turkish patients with cancer, and Gardiner et al. study (2013) on low-income racially diverse patients in the USA confirm the impact of health literacy on the use of complementary and alternative medicine (25, 29, 57, 58). Health literacy is a holistic structure that consists of functional, critical, perceptional, communication and decision-making skills and can influence medical decision-making (59), and strengthening health literacy helps make a fully informed decision about the use of complementary medicine. However, Bains et al. (15) examined adult patients referred to a primary care and outpatient clinic in the southwestern United States. They showed a significantly different relationship between health literacy and the use of complementary medicine in terms of race, so that adequate health literacy among whites was significantly associated with increased use of complementary medicine, while adequate health literacy among African Americans had no effect on the use of complementary medicine (15). Smith et al. (17) showed no significant relationship between health literacy and the use of complementary medicine among Australian patients (17). Differences between the mentioned studies and the current research were due to cultural differences, high use of nonprescription drugs, different information sources about complementary medicine, and even the use of different health literacy tools.

The relationship between health literacy and CAM may help health providers determine which patients are more likely to use CAM, understand how patients of varying health literacy levels relate to allopathic care, direct educational interventions related to CAM, as well as help researchers design CAM clinical trials that take into account the health literacy of participants (58).

Based on the study results, there was a significant relationship between insurance and the use of complementary and alternative medicine, but there was no relationship between other variables and the use of complementary and alternative medicines. According to the results, insured individuals used complementary and alternative medicine more than uninsured ones. According to Radwan et al. (39) in the UAE, insured patients used complementary medicine more (39). The sampling was done in clinics and most of the patients were insured. However, Naja et al. (60) in Lebanon showed that uninsured patients showed a greater desire to use complementary medicine (60). The reason for this difference may be the low cost of complementary medicine compared to modern medicine (39). The findings of other studies also showed no relationship between education, age, and the use of traditional and complementary medicine (13, 61), but Eshag-Hosseini and Khorasani (62) reported a significant relationship between the use of complementary medicine, the child’s age and mother’s education (62). The reason for the difference in the results may be attributed to the difference in the age groups studied. Children under 10 years of age participated in Hosseini’s study. Usually, parents at a younger age try to use herbal medicines for their children, except for special cases that require the use of chemical medicines due to the side effects of chemical medicines.

## Limitations

This study had several limitations. Some patients did not want to complete the questionnaires due to their unfavorable mental and physical conditions. Other reasons were unsuitable environment for interviewing with patients, COVID-19 epidemic and the presence of patients with COVID-19, the crowdedness of the medical centers and the lack of a quiet place for the patients to talk easily. Since the study participants were those who visited the clinics, this study did not include all chronically ill outpatient cases, so results should be generalized with caution. Furthermore, as all clinics have the same conditions in our setting, we did not compare the characteristics of participants referring to different clinics. However, this issue might have affected the interpretation of data; therefore, data should be interpreted with caution. In addition, there was a possibility of recall bias in the results because the samples were asked to report the level and type of complementary medicine they used in the last year.

## Conclusion

The results of the present study showed that the mean use of complementary and alternative medicine in the last year was low. Therefore, increasing the awareness of chronically ill outpatient cases about types of complementary and alternative medicine can be effective in expanding the use of complementary medicine among them. As healthcare workers have more contact with patients, their information about the benefits and risks of using complementary and alternative medicine can be helpful. In addition, the total mean scores of health literacy and its dimensions in chronically ill outpatient cases were borderline, so that health literacy predicted the use of complementary and alternative medicine. Health education and promotion programs may be useful for improvement of health literacy in the community.

### Implications and policy recommendations

Healthcare workers can improve health literacy by specialized educational interventions. It is hoped that future researches address interventions related to health literacy, train chronically ill outpatient cases to increase their health literacy about their disease, examine the relationship between health literacy and the use of drugs prescribed by doctors, and measure how patients’ health literacy affects their medication adherence.

## Data availability statement

The raw data supporting the conclusions of this article will be made available by the authors, without undue reservation.

## Ethics statement

The studies involving human participants were reviewed and approved by Kerman University of Medical Sciences. The patients/participants provided their written informed consent to participate in this study.

## Author contributions

MD, MM-R, and LL designed the study and wrote the manuscript. MD and LL contributed to the study design, they provided critical feedback on the study and statistical analysis, and inputted to the draft of this manuscript. MR, BG-n, and MM-R collected data. All authors have read and approved the final manuscript.

## Conflict of interest

The authors declare that the research was conducted in the absence of any commercial or financial relationships that could be construed as a potential conflict of interest.

## Publisher’s note

All claims expressed in this article are solely those of the authors and do not necessarily represent those of their affiliated organizations, or those of the publisher, the editors and the reviewers. Any product that may be evaluated in this article, or claim that may be made by its manufacturer, is not guaranteed or endorsed by the publisher.
